# Editorial: The Mechanisms of Insect Cognition

**DOI:** 10.3389/fpsyg.2019.02751

**Published:** 2019-12-05

**Authors:** Lars Chittka, Martin Giurfa, Jeffrey A. Riffell

**Affiliations:** ^1^School of Biological and Chemical Sciences, Queen Mary University of London, London, United Kingdom; ^2^Research Center on Animal Cognition, Center of Integrative Biology, CNRS - University Paul Sabatier - Toulouse III, Toulouse, France; ^3^Department of Biology, University of Washington, Seattle, WA, United States

**Keywords:** brain, cognition, computation, learning, memory, neuroscience

Insects have miniature brains, but recent discoveries have upturned historic views of what is possible with their seemingly simple nervous systems (Giurfa, 2013). Phenomena like tool use (Loukola et al., 2017; Mhatre and Robert), face recognition (Chittka and Dyer, 2012; Avarguès-Weber et al.), numerical competence (Skorupski et al., 2018; Howard et al., 2019), and learning by observation (Leadbeater and Dawson, 2017) beg the obvious question of how such feats can be implemented in the exquisitely miniaturized bio-computers that are insect brains. Bringing together researchers from insect learning psychology and neuroscience in a common forum in this Research Topic was envisaged to inject momentum into this dynamic and growing field. In selecting our authors, we have deliberately chosen not to focus on seniority and pontification—we have made a concerted effort to recruit junior authors, as well as scientists from diverse countries (of 17 nations) including some where current governments have erected substantial obstacles to basic research.

Many remarkable behavioral feats of insects concern their navigation. Examples include insects that can memorize multiple locations that are many kilometers apart (Collett et al., 2013), and some can find and remember efficient routes to link multiple destinations (Lihoreau et al., 2012). Understanding the neural underpinnings of such spatial memory feats is a fascinating endeavor, and this field forms thus one of the key areas of our Research Topic.

Some of the champions of insect navigation are ants which can find their way over long distance in either cluttered environments, or, as in some desert species, in featureless terrain (Freas and Schultheiss). Nocturnal bull ants can even make adaptive use of the third dimension—when they are viewing the familiar scenery from above, while climbing on trees (Freas et al.). Using modeling, Le Moël et al. demonstrate that a tiny area of the brain of ants and other insects, the central complex, can integrate landmark and vector memories as well as a sun compass (which requires knowing the time of day). Because of its role in integrating external input with internal simulation of the environment, the central complex has recently been pinpointed as a possible seat of consciousness in insects (Barron and Klein, 2016). An argument for this notion comes from the work of Libersat et al. on parasitoid wasps that highjack their insect hosts' brains to disable normal behavior, manipulating them in such a way as to benefit the parasites. The wasps sting their hosts into a brain area around the central complex, which aborts all self-initiated behavior in the hosts.

But Hymenoptera (bees, ants, and wasps) are not the only insects that are excellent navigators. Pomaville and Lent, working with paradigms such as split-brain cockroaches, discover that these insect have multiple navigation systems in their brains, which are integrated into a coherent whole. The kissing bug displays considerable versatility in spatial avoidance learning (Minoli et al.). While in some cases, innate responses to certain stimuli are unaffected by experience, the bugs can learn to associate stimuli in a wide variety of sensory modalities as predictors of aversive stimuli. Indeed, using *Drosophila* as a model, Gorostiza shows just how outdated the idea of insects as reflex machines really is: almost all behavior routines previously thought to be innate, also have cognitive components. This is even the case for some spatial behavior routines that were previously thought to be entirely governed by instinct—the construction of hexagonal comb structures in bees. From the exploration of the historic literature— Gallo and Chittka found experimental evidence, over 200 years old and buried in largely forgotten literature, showing that honeybees might display a mental planning ability in their comb constructions (Huber, 1814).

Several papers of the volume show how complex cognitive functions can be implemented with clever behavioral shortcuts, using minimal neural circuitry: e.g., the Mhatre and Robert shows the tricks by which insects can manufacture tools, Avarguès-Weber et al. discover the psychological strategies by which some insects can recognize faces, and Guiraud et al. determine how bees can solve a visual concept learning tasks successfully but in a manner totally alien to humans. Insects also display “personality”—as evidenced when each individual's emphasis on speed and accuracy when choosing between foraging options under predation threat (Wang et al.), and (Tomasiunaite et al.) demonstrate how these often complex and individual behaviors can be quantified using automated computer vision methods.

A recurrent theme of the volume is that complexity can arise even from “simple” associative learning, similarly to mammals. When *Drosophila* larvae learn to discriminate rewarded form non-rewarded stimuli, they not only learn to search for the rewarded stimulus, but also to avoid the non-rewarded stimulus, revealing the existence of multiple simultaneous memory traces arising from the same learning (Schleyer et al.). The Prediction Error Theory (Rescorla and Wagner, 1972) originally proposed for mammals and stating that the discrepancy between the actual and the predicted reward is a main determinant of learning, also applies to cricket learning (Mizunami et al.). Similarities also occur at the molecular level as the signaling pathways leading to the formation of long-term memory in crickets share attributes with those of mammals (Matsumoto et al.).

Several contributions focus on odor learning, for example in a South American bumblebee species (Palottini et al.). *Drosophila* larvae, after being trained to discriminate two odors, reveal the principles of odor mixture processing based on their responses to combinations of the two odors trained (Chen et al.). Learning of multisensory stimuli is studied by Mansur et al., who adopted so-called patterning protocols, which explore insects' capacity to treat stimulus compounds as being different from the simple sum of their components.

The importance of nutritional state on learning has received considerable attention in mammals. In honeybee olfactory learning, best performance is achieved by a diet rich in essential fatty acids, as long as the omega-6:3 ratio is not high (Arien et al.). In bees, lateralization of odor processing occurs with respect to the two antennae (Frasnelli et al., 2012). Baracchi et al. extend this analysis to the processing of the sucrose reward and find a right hemisphere dominance, adding a further dimension to brain lateralization in bees.

The specific brain regions, neural circuits and genes allowing behavioral complexity in insects are explored in several contributions. The role of inhibitory signaling via GABAergic neurons in various brain regions of moths, crickets and bees is highlighted by Ai et al. The mushroom bodies are the main neural substrate for memory storage and retrieval. Focusing on the learning of a specific time of the day as predictor of food reward, Shah et al. show that the expression of the immediate early gene Egr-1 of bee mushroom bodies is regulated by the circadian clock. Suenami et al. show that types of mushroom body cells can be distinguished based on protein and gene-specific differences.

Insects are able to produce highly sophisticated behavior—as highlighted by our issue—using fewer neurons than vertebrate brains. The possibility of identifying specific neural architectures as the basis for some forms of cognitive processing has a unique potential for the development of research on artificial intelligence and robotics, and possibly for the understanding of the human brain. Psychologists and neuroscientists, as well as engineers and computer scientists, are thus beginning to appreciate insects as a model system for examining how intelligence can be achieved with miniature nervous systems. The exploration of the brain mechanisms mediating complex cognition in insects is key to understanding the very nature of intelligence—in all animals, not just insects. Finally, the insect apocalypse (mass extinctions as a result of habitat destruction, pesticide use, and climate change) is global news. Insects have not just important ecosystem functions but are vital to human food security—e.g., one third of the food we consume (most fruits and vegetables) are directly contingent upon insect pollination. Understanding their cognition and the richness of their perceptual worlds adds a new slant to their conservation and research ethics.

## Author Contributions

All authors listed have made a substantial, direct and intellectual contribution to the work, and approved it for publication.

### Conflict of Interest

The authors declare that the research was conducted in the absence of any commercial or financial relationships that could be construed as a potential conflict of interest.
